# Harnessing Social Media to Explore Youth Social Withdrawal in Three Major Cities in China: Cross-Sectional Web Survey

**DOI:** 10.2196/mental.8509

**Published:** 2018-05-10

**Authors:** Lucia Lin Liu, Tim MH Li, Alan R Teo, Takahiro A Kato, Paul WC Wong

**Affiliations:** ^1^ Department of Social Work and Social Administration Faculty of Social Sciences The University of Hong Kong Hong Kong China (Hong Kong); ^2^ Department of Rehabilitation Sciences The Hong Kong Polytechnic University Hong Kong China (Hong Kong); ^3^ VA Portland Health Care System Health Services Research & Development Center to Improve Veteran Involvement in Care Portland, OR United States; ^4^ Department of Psychiatry Oregon Health & Science University, Portland, OR United States; ^5^ School of Public Health Oregon Health & Science University and Portland State University Portland, OR United States; ^6^ Department of Neuropsychiatry Graduate School of Medical Sciences Kyushu University Fukuoka Japan

**Keywords:** adolescent, social withdrawal, hikikomori, youth social issues, Web survey, China

## Abstract

**Background:**

Socially withdrawn youth belong to an emerging subgroup of youth who are not in employment, education, or training and who have limited social interaction intention and opportunities. The use of the internet and social media is expected to be an alternative and feasible way to reach this group of young people because of their reclusive nature.

**Objective:**

The aim of this study was to explore the possibility of using various social media platforms to investigate the existence of the phenomenon of youth social withdrawal in 3 major cities in China.

**Methods:**

A cross-sectional open Web survey was conducted from October 2015 to May 2016 to identify and reach socially withdrawn youth in 3 metropolitan cities in China: Beijing, Shanghai, and Shenzhen. To advertise the survey, 3 social media platforms were used: Weibo, WeChat, and Wandianba, a social networking gaming website.

**Results:**

In total, 137 participants completed the survey, among whom 13 (9.5%) were identified as belonging to the withdrawal group, 7 (5.1%) to the asocial group, and 9 (6.6%) to the *hikikomori* group (both withdrawn and asocial for more than 3 months). The cost of recruitment via Weibo was US $7.27 per participant.

**Conclusions:**

Several social media platforms in China are viable and inexpensive tools to reach socially withdrawn youth, and internet platforms that specialize in a certain culture or type of entertainment appeared to be more effective in reaching socially withdrawn youth.

## Introduction

*Hikikomori*, a form of pathological social withdrawal behavior that was first identified in Japan, describes youth and young adults who largely become recluses (mainly in their parents’ homes) and do not engage in education, employment, or training for months or years [1]. Their *hidden* or *non-engaged* behavior [2-6] makes studying *hikikomori* an extremely challenging research topic.

The most common research recruitment methods to identify young people in social withdrawal include referrals from mental health professionals [7-9], parents [10], and household surveys [11]. However, because of the small sample sizes of referrals and the low cost-efficiency of household surveys, researchers have suggested that alternative recruitment methods are needed to expand our understanding of this emerging worldwide youth phenomenon [1,3,4,7,12].

Wong and colleagues [13], for instance, were one of the very few investigators to adopt an alternative study methodology to engage potentially withdrawn young people. They used a telephone survey to study potentially withdrawn individuals in Hong Kong. In their study, a sample of 80,000 mobile numbers was randomly generated and contacted. They finally contacted 2854 eligible individuals, 1010 of whom completed the telephone survey. Of these, 70 individuals reported spending most of the day and nearly every day at home and persistently avoiding social situations (such as going to school or working) and social relationships (such as friendships and contact with family members). The main limitations of their study included lack of a population-based representative sample and a small sample of individuals who could be considered socially withdrawn. However, at that time, telephone survey was the least costly and most feasible method to study this youth phenomenon in Hong Kong. With the overall cost of HK $110,000 (around US $14,066.5), the cost per participant is HK $108.91 (US $13.93), which is derived by dividing the total number of completed surveys by the sum of total personnel (excluding the research team members’) cost and administrative costs.

Despite the rising concerns of scholars in mainland China about the existence of youth in social withdrawal [14,15], there are, as far as we know, no empirical studies that have been conducted on this emerging youth issue. Only a few case studies of potential socially withdrawn youths have been reported by youth social workers [15]. It is observed that less-developed social welfare services, coupled with low awareness of this youth issue among the public and even social service professionals, are some of the underlying barriers to launching a large-scale investigation of this population. Therefore, there is a need to explore innovative research approaches to reach a wider youth population. The use of the internet for data collection, which has been proved efficient in the West, could become a new recruitment strategy in China.

According to a recent report of the prevalence of the internet in China, by the end of December 2015, the total number of internet users in China had reached 688 million, 75.1% of whom were aged between 10 and 39 years [16]. Beijing, Shanghai, and Guangdong province were the top 3 areas with internet use rates above 70%. Sina Weibo and WeChat are now 2 of the most popular social media sites in China. Akin to a hybrid of Twitter and Facebook, Weibo combines the functions of a microblog and a social networking site and had accumulated 282 million monthly active users (MAUs) and 126 million daily active users as of June 2016 [17]. According to the annual report by the Chinese Academy of Social Science, as of 2014, the population aged between 10 and 39 years accounted for 78.7% of microblog users, and 82.2% were users of Sina Weibo [18]. The Chinese instant messaging program WeChat had attracted 806 million MAUs as of June 2016 [19]. With its group chat function and public accounts for individuals and organizations, WeChat has become a top platform of communication in China’s Web-based communities. Many social networking websites that have more specific focuses and target groups (eg, douban website for its focus on reviews of cultural products and activities, renren website for students to reconnect with old school friends, and Wandianba for people who are interested in video games and board games) also attract large numbers of users [20].

Given the ubiquity of the internet and social media among young people in China and the previous successful experience of Chinese scholars who recruited more than 1000 Weibo users to complete their survey on mental health issues (eg, [21]), we were interested in experimenting with various Web-based means to facilitate the administration of a research survey among young people in China. The main aim was to identify recruitment methods that are feasible and appropriate for use in a future study of pathological social withdrawal among young people in China to expand our understanding on this emerging but methodologically challenging issue in many countries.

## Methods

### Study Population

The recruitment area for the survey covered 3 major metropolitan cities in mainland China: Beijing, Shanghai, and Shenzhen (a major city in Guangdong Province). According to the 2010 census data, the population aged between 10 and 39 years in the 3 cities is 28,417,551, accounting for 53.6% of their overall population. A cross-sectional open Web survey was conducted from October 2015 to May 2016 to identify and reach our target population, ie, youth in social withdrawal.

### Recruitment

The survey was created and stored using a Chinese survey platform called Sojump, which is operated by Changsha Xingran Information Technology Company and is one of the biggest survey platforms in China. The survey host provided a unique link to the survey, and its system helped to block attempts by respondents with the same internet protocol (IP) address or the same electronic device to fill out the survey more than once. After completing the survey, respondents were able to review their answers and were reminded to finish unanswered mandatory items before submission.

Three recruitment methods were experimented sequentially. They included (1) Weibo, (2) WeChat group and microblog, and (3) an online game website.

#### Method 1

Weibo was chosen as the main social media platform to administer the survey. The procedure was as follows:

1. An official Weibo account was created for the research team to post invitations and information related to the survey
2. Weibo’s paid advertising service was adopted to send out survey invitations to Weibo users who met the following inclusion criteria:
  - IP addresses in Beijing, Shanghai, and Shenzhen
  - registered age on Weibo is between 13 and 39 years.

Material incentives were adopted to increase the survey response rates. Once the respondents finished the questionnaire, they were given a chance to enter a lottery for a CNY ¥500 (US $77.44) cash coupon. Only the winner was asked to leave the mobile phone number and be further contacted. As each Weibo account is tied with 1 mobile phone number and the survey information will only be pushed to each eligible user once at a random time point, this has reduced the chance of repeated attempts by same participants by using different Weibo accounts or electronic devices.

#### Method 2

Upon monitoring the effectiveness of Weibo in reaching the target population, to compensate for the low response rate, the research team also advertised the survey via WeChat with different approaches:

1. The survey information was sent to 2 WeChat groups: the group of the first author’s college alumni (with 61 members), most of whom reside in Beijing and Shanghai and are working in domains related to education, and a group of strangers who joined a free distance course on psychotherapy arranged by a training center (with 98 members)
2. Personal microblog on WeChat: through a professor at a renowned university in Shanghai, 2 students majoring in public health posted the survey link on their microblogs on WeChat; they attracted 18 responses within a 2-week period.

#### Method 3

With the permission of the administrator of 1 popular online game website, we were able to advertise the survey on Wandianba website [22] among its more than 8000 registered members. Members in these groups were allowed to forward the survey link to people they knew. The respondents recruited from these approaches could enter the “red envelope lucky draw” (a function developed by WeChat), and each respondent could win a random amount of “lucky money” up to CNY ¥10. A summary of the Web survey details according to the Checklist for Reporting Results of Internet E-Surveys guidelines [23] is provided in Multimedia Appendix 1. The survey invitation and post for different platforms is shown in Multimedia Appendices 2 and 3.

### Ethical Considerations

The respondents’ consent to participate in the study was obtained online. They were required to read and approve an informed consent form before proceeding to the questionnaire. Assent from adolescent participants was collected with the same consent process described above. The information sheet of the study was designed at the reading level of a 13 year old.

To limit response bias, the nature of the study was introduced in a general sense as concerning youth and internet use. The questions in the survey pertain to this general introduction, and no deception is involved throughout the research. At the end of the questionnaire, a message containing contact information and numbers for crisis intervention hotlines of local nongovernmental organizations that offer mental health services in the 3 cities was provided to the respondents to encourage them to seek help if they felt distressed upon completing the questionnaire.

### Data Security and Confidentiality

Because sensitive information such as risk behavior was collected in the survey, measures were taken to ensure data security throughout the research. During the data collection process, only the principal investigator had access to the account on the website that hosted the survey. The survey host also followed the cyber security protocol to prevent data leakage. Once data collection was finished, all of the research data were coded and stored on password-protected drives. Ownership of and access to the data were restricted to the research team. The study obtained ethical approval from the Human Research Ethics Committee for Non-Clinical Faculties at the University of Hong Kong (Ref# EA1508009).

### Measurements

#### Social Withdrawal Symptoms as Dependent Variables

This study used a modified version of the definition of *hikikomori* proposed by Teo [1,3]. In brief, the survey inquired about (1) physical isolation or withdrawal to a particular place, (2) lack of social connectedness and interaction, and (3) duration of social withdrawal. For this study, we used a 3-month rather than 6-month duration of symptoms because recent research has suggested similar characteristics of individuals with a shorter duration of symptoms. Participants were assigned to the withdrawal group (only meeting criteria 1 and 3: staying at home almost every day for more than 3 months), the asocial group (only meeting criteria 2 and 3: persistently avoiding social interaction for more than 3 months), or the *hikikomori* group (meeting all 3 criteria). Participants who did not meet any of the 3 criteria were assigned to the comparison group.

The independent variables comprised the demographics, individual, social, and parental domains of participants, which are the major dimensions discussed in youth social withdrawal research [4].

#### Demographics

The respondents were invited to provide their profile information, including age, gender, highest education level, marriage status, and parental information.

#### Internet Social Capital Scales

The scales were used to measure the quantity and quality of respondents’ social networks both online and offline. They comprise 2 parallel 5-point Likert scales to test respondents’ online and offline social capital. In each scale, 20 items measure the respondents’ bonding and bridging social capital [24].

#### Common Measurements for Social Media Advertising

Weibo uses metrics similar to dominant advertising services (eg, Facebook) to show the popularity of each post, including impressions, clicks, reach, and click-through rate: (1) an impression is defined as a single time an ad is shown to a user regardless of whether the user clicks on the ad, (2) a click is when a user clicks a link in an ad, (3) reach is the number of unique people who received impressions of an ad, and (4) click-through rate is calculated by the number of clicks of a received ad divided by the number of impressions.

### Statistical Analyses

Descriptive statistics for the continuous variables were illustrated by means and standard deviations, whereas categorical variables were shown by numbers and percentages. Fisher exact test and the Mann-Whitney-Wilcoxon test were used to compare the categorical and continuous variables, respectively, between the comparison group and the withdrawal group, the asocial group, and the *hikikomori* group.

## Results

### Overall Recruitment

**Figure 1:**
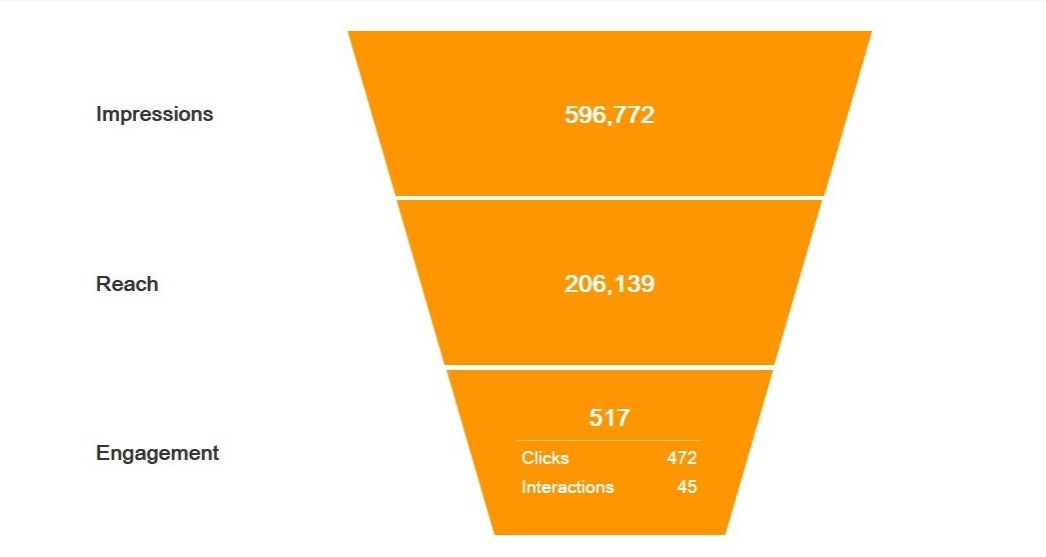
Effectiveness funnel of Weibo.

The Web-based questionnaire was administered at 3 sites (ie, Weibo, WeChat, and Wandianba). According to the statistics generated by Weibo, the survey was exposed 596,772 times to targeted users and reached 206,139 users. Of these, 517 (click-through rate=0.087%) either clicked on the survey link or forwarded the post to others. However, only 85 completed the survey via Weibo (Figure 1). The posts on WeChat groups yielded 43 responses, and Wandianba attracted 9 responses (Table 1). The survey host recorded a total of 192 visits with independent IPs to the survey, making the completion rate 71.4%. The survey ultimately collected a total of 137 responses within a period of 7 months.

### Costs of Social Media Advertising

Advertising on Sina Weibo is managed on the internet, and pricing is based on a bidding system. There are 2 ways to bid for the target group among advertisers with the same aim: to pay per 1000 impressions and to pay per click. During the 7-month advertising on Sina Weibo, we tried both methods of advertisement with different bidding prices based on factors such as the response rate and the number of advertisers opting for the same target group in different time periods. On the first day of advertisement, we chose to pay by impressions. A price of CNY ¥10.8 (around US $1.67) per 1000 impressions was set; however, there were no responses. Thus, starting on October 20, 2015, the bidding method was changed to pay by click starting with prices ranging from CNY ¥10 per click to CNY ¥20 per click (the average price was CNY ¥12.5). It was found that the bidding price did not significantly influence the response rate. However, the number of visits to the recruitment page and the responses to the survey decreased remarkably during the seasonal marketing campaign of the e-commerce website (taobao, the largest e-commerce website in China).

Ultimately, the cost of the 7-month campaign on Sina Weibo was CNY ¥3300 (US $511.07), and the overall service charges (including monthly fee, administration fee for the lucky draw, and distribution of cash rewards) from the survey host (Sojump) and cash rewards (CNY ¥1000 in total) were CNY ¥3128 (US $484.44). Thus, the average cost to reach a participant who completed the survey through Sina Weibo was CNY ¥46.92 (US $7.27).

**Table 1:** Survey administration procedure and distribution of responses.

Media	Administration period	Additional remarks	Number of responses
Weibo	Oct 19 to Oct 20, 2015	Charged by per 1000 impressions; because none of these impressions were transferred into responses, the advertising strategy was changed to the interaction rate from Oct 20 onwards	85
	Oct 20, 2015, to May 20, 2016	Charged by per click of the link	
WeChat groups	Feb 2 to Feb 22, 2016	During the period of Chinese New Year, the survey information was posted on several WeChat groups.	43
Wandianba website [22]	Apr 1 to Apr 30, 2016	The research team posted the survey information on several internet communication platforms that appeal to young people, including Mop, Hupu, Tianya, Baidu Tieba, and Wandianba. However, except for Wandianba, where the administrator approved the attempt to advertise the survey, other platforms immediately banned and deleted the messages posted	9

Except for the incentives in the form of cash coupons of random amounts, there was no additional cost for advertisement via WeChat group and Wandianba. As compared with the average cost per participant of a telephone survey targeting the same population, the cost is relatively lower and could be a viable method in future studies.

### Preliminary Findings on the Population of Hikikomori in Mainland China

In total, 137 participants completed the survey. Of the 137 responding participants, 85 were recruited through Weibo (over 7 months at a cost of around US $500), 43 from WeChat (over 20 days at no cost), and 9 from Wandianba website (over 30 days at no cost). Among the 137 participants, 108 (78.8%) did not show evidence of social withdrawal symptoms and therefore were classified as the control, or comparison, group. The remaining 29 (21.2%) participants had symptoms of social withdrawal and were classified as follows: the 13 (9.5%) who indicated that they stayed at home nearly every day for a period of more than 3 months were categorized as the withdrawal group; the 7 (5.1%) participants who reported that they avoided most social contact for a period of more than 3 months were categorized as the asocial group; and the 9 (6.6%) participants who met both the withdrawal and asocial criteria for more than 3 months were categorized as the *hikikomori* group. Both the withdrawal group and the *hikikomori* group were more likely than the comparison group to access the Web-based survey through Wandianba but were less likely to assess the survey through WeChat (Table 2). The *hikikomori* group was more likely than the comparison group to access the Web-based survey through a computer.

**Table 2:** Comparison of access to the survey categorical and continuous variables of the classified participants using Fisher exact test and Mann-Whitney-Wilcoxon test, respectively.

Online behaviors		Comparison group	Withdrawal group	*P* value	Asocial group	*P* value	*Hikikomori* group	*P* value
**Mode of survey administration**								
	Phone, n (%)	83 (76.9)	10 (77)	>.99	4 (57)	.36	4 (44)	.047
	Computer, n (%)	25 (23.1)	3 (23)		3 (43)		5 (56)	
**Platform to survey**								
	Weibo, n (%)	64 (59.2)	10 (77)	.04	6 (86)	.42	5 (56)	.007
	WeChat, n (%)	40 (37.0)	1 ( 8)		1 (14)		1 (11)	
	Wandianba, n (%)	4 ( 3.7)	2 (15)		0 ( 0)		3 (33)	
	Time spent on questionnaire (in seconds), mean (SD)	757.80 (768.52)	644.08 (165.58)	.91	870.00 (1031.51)	.29	835.33 (653.50)	.67
**Number of online friends**								
	Weibo, mean (SD)	94.60 (212.40)	24.09 (55.07)	.02	7.60 (9.24)	.07	57.17 (119.46)	.37
	WeChat, mean (SD)	163.40 (313.38)	57.36 (92.56)	.003	104.00 (42.78)	.97	27.33 (26.10)	.004
	QQ^a^, mean (SD)	196.89 (173.86)	155.55 (182.07)	.17	136.00 (72.32)	.62	127.29 (210.30)	.04
	Frequency of contacting online friends, mean (SD)	1.84 (1.19)	1.77 (0.83)	.76	1.14 (0.38)	.13	2.56 (1.42)	.09

^a^QQ: a widely used instant messaging software in China.

**Table 3:** Comparison of sociodemographics categorical variables of the classified participants using Fisher exact test.

Characteristics		Comparison group, n (%)	Withdrawal group, n (%)	*P* value	Asocial group, n (%)	*P* value	Hikikomori group, n (%)	*P* value
**Gender**								
	Male	43 (39.8)	5 (38)	>.99	2 (29)	.70	6 (67)	.16
	Female	65 (60.1)	8 (62)		5 (71)		3 (33)	
**Age in years**								
	<18	13 (12.3)	6 (46)	.02	0 ( 0)	.47	1 (11)	>.99
	18-24	56 (52.8)	5 (38)		5 (83)		5 (56)	
	>24	37 (34.9)	2 (15)		1 (17)		3 (33)	
**Education level**								
	Above secondary Form 3	91 (84.3)	8 (62)	.06	7 (100)	.59	8 (89)	>.99
	Form 3 or below	17 (15.7)	5 (38)		0 (0)		1 (11)	
**Marital status**								
	Single	82 (79.6)	8 (67)	.29	6 (100)	.59	6 (67)	.40
	Married/cohabitating	21 (20.4)	4 (33)		0 (0)		3 (33)	
**Family structure**								
	Both parents	95 (88.0)	10 (77)	.38	6 (86)	>.99	8 (89)	>.99
	Others	13 (12.0)	3 (23)		1 (14)		1 (11)	
**Employment status**								
	Student	40 (37.0)	8 (62)	.01	3 (43)	>.99	3 (33)	.10
	Employed	64 (59.3)	3 (23)		4 (57)		4 (44)	
	Unemployed	4 (3.7)	2 (15)		0 (0)		2 (22)	
**Visited psychiatric hospital or clinics**								
	Yes	3 (2.8)	0 (0)	>.99	0 (0)	>.99	2 (22)	.047
	No	105 (97.2)	13 (100)		7 (100)		7 (78)	

**Table 4:** Comparison of social capital continuous variables of the classified participants using the Mann-Whitney-Wilcoxon test.

Social capital	Comparison group, mean (SD)	Withdrawal group, mean (SD)	*P* value	Asocial group, mean (SD)	*P* value	Hikikomori group, mean (SD)	*P* value
Online bonding	25.05 (6.10)	24.69 (5.54)	.79	26.71 (6.90)	.70	28.56 (5.94)	.16
Online bridging	32.34 (7.81)	36.85 (8.31)	.07	35.43 (9.69)	.13	36.33 (8.49)	.18
Offline bonding	35.57 (5.81)	33.62 (6.41)	.12	37.43 (4.79)	.64	32.89 (5.62)	.20
Offline bridging	35.81 (7.08)	36.15 (8.44)	.97	38.00 (6.95)	.43	30.56 (8.93)	.07

The withdrawal group had fewer online friends on Weibo and WeChat than the comparison group, whereas the *hikikomori* group had fewer online friends on WeChat and QQ (a widely used instant messaging software in China) than the comparison group. The withdrawal group was younger and included more students (62%, 8/13) and unemployed individuals (15%, 2/13) than the comparison group (Table 3). The *hikikomori* group was more likely to have visited psychiatric hospital or clinics than other participants. The withdrawal group had more online bridging social capital than the comparison group, whereas the *hikikomori* group had less offline bridging social capital than the comparison group (Table 4). There was no significant difference between the asocial group and the comparison group in terms of the studied variables.

## Discussion

### Principal Findings

As far as we know, this is the first quantitative study to evaluate *hikikomori* and social withdrawal behavior among young people in China. Given the high penetration of social media among young people in China, we explored the feasibility of using various social media platforms to recruit young people to participate in our Web survey.

The response from the 3 recruitment methods shows very interesting patterns. Although Weibo helped in recruiting the largest portion of participants, it took 7 months to reach that number, whereas it took only 20 days to recruit 43 participants through personal contacts using WeChat. Although it took a month to recruit 9 participants at Wandianba, 3 of those participants were *hikikomori*. In other words, online gaming sites merit further exploration as a venue to identify socially withdrawn youth in China.

Despite the small dataset, we identified 13 physically withdrawn youths, 7 asocial youths, and 9 *hikikomori*, making the percentages among the overall participants 9.5% (13/137), 5.1% (7/137), and 6.6% (9/137), respectively. This finding indicates that we reached a high percentage of individuals at risk of social withdrawal behavior and that we appeared to have targeted the correct group of people through our various social media recruitment methods. Although the data collected from convenient/snowball sampling could be biased, it seems that the use of WeChat and social networking sites that cater to particular user groups are especially effective in reaching over 6% of potential socially withdrawn youth in China, with reference to the prevalence rate in modern society of around 2% [4,13]. This exploratory study also consolidates our experiences with online questionnaire administration in China.

### Challenges Awaiting to be Addressed

First, because of the tight internet censorship of the Chinese Government, to avoid being shut down, internet content providers will self-censor their content [25]. For example, there were several words in our questionnaire (eg, names of illicit drugs) that were considered sensitive, and the research team was asked by the survey host (Sojump) to change the wording or the display of the words. On the basis of this incident, it is estimated that similar surveys distributed through official avenues such as schools and residential committees are very likely to be sanctioned.

Second, following the auction principle set up by Weibo, the research team acted as a bidder offering a price that represents the maximum willingness to pay for an advertisement. The team had to compete with other commercial advertisers to gain audiences. However, because social media platforms are pervasively used for commercial purposes, the cost of administering the survey has increased. In addition, there are several waves of commercial campaigns in China, such as the big sales on November 11 (known as “Single’s Day”), December 12 (known as “Double Twelve”), and Chinese New Year’s Eve, and responses stagnated during these marketing periods. Therefore, to make the advertisement cost-effective, it is important to find a strategy to buffer the impact of these commercial campaigns on response rate.

### Suggestions for Future Research

Our findings suggest 4 approaches to secure a higher response rate for Web-based surveys in China. First, the use of a prominent Sina microblog account to promote the survey was especially conducive to data collection. In our study, we created a new account to introduce the information of the Web survey, which limited exposure to the targeted population and raised administration costs. In contrast, Guan et al [21] used a celebrity account to promote their survey and easily gathered more than 1000 responses within a shorter period. Therefore, we highly recommend that future research should use a microblog account with a greater fan base.

Second, the length of the survey should be within the participants’ attention spans. Although several measures were used to shorten the survey, such as using a short version of the scale and adopting skip logic to ensure that certain scales were only shown to respondents who met the criteria of the target population, the average time to complete the survey was 15 min. Although the duration is not unbearable to general participants, it is still a challenge to sustain participants’ attention; 192 participants with independent IP addresses entered the survey but only 137 of them completed it, resulting in a completion rate of 72.4%.

Third, the use of scales and the presentation of the survey should be intelligible to the targeted population. Comments from participants left on Wandianba regarding the survey recorded several complaints about the format of the questions. To individuals without much exposure to psychological measurement, many questions in the survey seemed redundant, such as the same set of questions asked regarding different variables such as online social capital and offline social capital. Researchers can also consider using application programming interfaces provided by social media to collect users’ publicly available digital records. For instance, users’ profiles and messages can be extracted to show their psychological [21,26] and behavioral features [27].

Fourth, the snowball sampling method we used on the Wandianba website has helped us to reach 9 potential severely withdrawn young people. This seems to be an effective recruitment method. In fact, this method is similar to a more advanced snowball sampling technique named as respondent-driven sampling. In respondent-driven sampling, a sample is collected using a chain-referral procedure, meaning that respondents are selected not from a sampling frame but from the social network of existing members of the sample. In addition, researchers keep track of who recruited whom and their numbers of social contacts, and then a mathematical model of the recruitment process weighs the sample to compensate for nonrandom recruitment patterns. This model is based on a synthesis and extension of 2 areas of mathematics, Markov chain theory and biased network theory, which were not part of the standard tool kit of mathematical sampling theory. The resulting statistical theory enables researchers to provide both unbiased population estimates and measures of the precision of those estimates [28]. This extends the realm within which statistically valid samples can be drawn, to include many groups of importance to public health, public policy, and arts and culture [29]. In the future, adopting the respondent-driven sampling method may be useful to recruit a bigger and more targeted sampling with a statistical weighting technique.

### Implication for Practice of Helping Professionals

The findings of the withdrawal group, the asocial group, and the *hikikomori* group suggested that they tend to converge on internet platforms that specialize in a certain culture or type of entertainment. For example, in our case, the gaming website Wandianba seems to appeal to socially withdrawn youth regardless of their withdrawal status. This feature indicates the necessity for social workers and other service providers to develop sufficient knowledge about popular youth culture and websites in their attempts to work with socially withdrawn youths [30]. This not only opens up possible channels to reach out to socially withdrawn youths but also serves as an approach to engage these young people.

Although it is found that online or cyber social capital can positively contribute to individuals’ well-being [31,32], no study has yet measured their social relationships and social networks online and how they affect young people’s social withdrawal behavior. Our study reveals an intriguing phenomenon in that young people in the asocial group did not avoid all kinds of social interaction. Moreover, by connecting with people from different backgrounds online, the withdrawal group could establish significant online bridging social capital. However, the *hikikomori* group might be prone to cut themselves off from weak ties, such as those with former colleagues and classmates, and as a result, offline bridging social capital cannot be built. The findings indicate the need to adopt a strength-based approach in working with different types of socially withdrawn youths. By examining and capitalizing on how those who work with socially withdrawn youths develop and maintain online social relationships with these young people, we may finally help them to figure out ways into the real social world.

### Limitations

Although internet use has reached nearly 50% in China [33], it clearly does not include everyone in some target audiences. Socioeconomic factors such as internet and smartphone accessibility limit the types of individuals who are exposed to a survey [34]. This clearly limits the audience reach. In addition, as a general limitation of the Web survey, the nonresponse rate increases the bias of estimators [35]. It is likely that the socially withdrawn youths who responded to the survey were those who were still motivated to connect with the outside world. Young people who totally cut themselves out from the social world might not be reached through this study. Moreover, studies that examine sensitive topics may not be as feasible in countries such as China because of internet censorship and a government-controlled media environment. This situation calls for a joint effort from mental health professionals, social workers, and psychiatrists to codevelop research and practices to assist the young people to reconnect with our society [36].

### Conclusion

As the first attempt to understand the population and situation of *hikikomori* in mainland China, this study has explored 3 different Web-based platforms in China (ie, 2 prevalently used social media Weibo and WeChat and 1 online gaming website) to reach youth in social withdrawal. We found that these platforms are viable and serve as inexpensive tools to reach socially withdrawn youth. It was also found that given the behavioral pattern of socially withdrawn youth, they tend to disengage from common social interaction, internet platforms that specialize in a certain culture or type of entertainment were more likely to reach such population.

## Appendix

Multimedia Appendix 1 Checklist for Reporting Results of Internet E-Surveys summary.

Multimedia Appendix 2 Survey invitation.

Multimedia Appendix 3 Survey message pages on Weibo.

## Abbreviations

IP: Internet protocol
MAUs: monthly active users
