# Supplementary material for: Harnessing Social Media to Explore Youth Social Withdrawal in Three Major Cities in China: Cross-Sectional Web Survey
Source: JMIR Ment Health. 2018 May 10;5(2):e34. doi: 10.2196/mental.8509 (PMC5968215; doi:10.2196/mental.8509)
Supplement: Multimedia Appendix 1 [file mental_v5i2e34_app1.pdf]

Appendix 1. CHERRIES Summary

Item Category	Checklist Item	Explanation
Design	Survey design	The target population was socially withdrawn youth in three major cities in mainland China (i.e., Beijing, Shanghai, and Shenzhen).
IRB approval and informed consent process	IRB approval	The study protocol was approved by the Human Research Ethics Committee for Non-Clinical Faculties at The University of Hong Kong.
	Informed Consent	Before proceeding to the content of the survey, potential participants were presented with a one-page introductory note including the research purpose, the identity of the investigator, the length of time of the survey, and the data storage method.
	Data protection	A commercial web survey provider, Sojump, was used. All of the information submitted by the respondents was encrypted. All of the data were hosted by Sojump, and only the principal investigator had access to the raw data.
Development and pre-testing	Development and testing	In September 2015, the survey was developed and tested among 14 current research students. Based on their comments, several items were refined (mainly to align the grammar and wording with the colloquial expressions of mainland Chinese). Changes were also made to address some technical issues reported by the students to allow the participants

		to pause and resume the survey from where they left off, to show a progress bar to inform participants of the percentage completed, and to break the questionnaire into several pages. We selected Sojump as the survey host because it could meet the abovementioned requirements.
Recruitment process and description of the sample having access to the questionnaire	Open survey vs. closed survey	The survey is an open survey, namely, young people who met the inclusion criteria have an equal chance to be exposed to the survey invitation.
	Contact mode	All of the initial contact with the potential participants was made on the Internet using three major social media sites: Sina Weibo, Wechat, and Wandianba.
	Advertising the survey	As stated above, the survey was advertised using the three social media sites, with Sina Weibo as the primary tool. Targeted users from these media were exposed to the same survey invitation (see appendix 1).
Survey administration	Web/E-mail	The survey was administered via the Internet.
	Context	Sina Weibo was chosen as the key platform to advertise the survey and was supplemented with two other social media sites (i.e., Wechat and Wandianba). Please refer to corresponding part of the manuscript for detailed descriptions of these platforms.

	Mandatory/voluntary	It is a voluntary survey.
	Incentives	All the participants were eligible to enter the lottery.
	Time/Date	The survey was conducted from October 2015 to May 2016.
	Randomization	No items or questionnaires were randomized.
	Adaptive questioning	Adaptive questioning was used. Based on participants' answers to certain questions (self-reporting of the condition and family background), they were directed to corresponding questions.
	Numbers of items	The questionnaire contains five scales.
	Number of screens	The whole questionnaire was broken into four online pages; however, due to skip logic, some respondents were presented with fewer than four pages of questionnaire.
	Completeness check	Each submitted response was checked for completeness.
	Review step	Respondents were able to review and change their answers before submission.
Response rates*	Unique site visitors	Through Sina Weibo, the survey advertisement was exposed to 596,772 users who met the inclusion criteria. However, there are no data on exactly how many users visit Wandianba, so we cannot estimate the

		number of unique site visitors.
	View rate	NA
	Participation rate	NA
	Completion rate	119 of 154 participants completed the survey, for a completion rate of 77.27%
Preventing multiple entries from the same individual	Cookies used	NA
	IP check	The IP address of each user's computer was used to identify potential duplicate entries from the same user. The setting was applied throughout the whole survey administration period. In cases in which duplicate entries were record, only the first entry was kept for analysis.
	Log file analysis	NA
	Registration	NA
Analysis	Handling of incomplete questionnaires	Only the completed questionnaires were analyzed.
	Questionnaires submitted with an atypical timestamp	NA

	Statistical correction	NA
