# Supplementary material for: Harnessing Social Media to Explore Youth Social Withdrawal in Three Major Cities in China: Cross-Sectional Web Survey
Source: JMIR Ment Health. 2018 May 10;5(2):e34. doi: 10.2196/mental.8509 (PMC5968215; doi:10.2196/mental.8509)
Supplement: Multimedia Appendix 2 [file mental_v5i2e34_app2.pdf]

Appendix 2. Survey invitation

Invitation on Weibo and Wandianba

Every generation has its own story to tell. You are cordially invited to join the web survey to show the spectrum of our lifestyle and get the chance to win $500 cash upon finishing the questionnaire. It only takes you around 10 to 15 minutes. Wanna try? Click here→survey weblink

Invitation on Wechat group

1. Every generation has its own story to tell. You are cordially invited to join the web survey to show the spectrum of our lifestyle. Upon finishing the questionnaire, you can enter to win a red packet worth up to $50. It only takes you around 10 to 15 minutes. Wanna try? Click here→survey weblink
2. https://sojump.com/m/11445370.aspx?from=singlemessage&isappinstalled=0

   Help! Folks, if you are currently aged 13-39, please click the link above to fill a questionnaire about youth lifestyle. Don't miss it—a surprise red packet awaits you upon completion.
