# Supplementary material for: Harnessing Social Media to Explore Youth Social Withdrawal in Three Major Cities in China: Cross-Sectional Web Survey
Source: JMIR Ment Health. 2018 May 10;5(2):e34. doi: 10.2196/mental.8509 (PMC5968215; doi:10.2196/mental.8509)
Supplement: Multimedia Appendix 3 [file mental_v5i2e34_app3.pdf]

Appendix 3. Survey message pages on Weibo

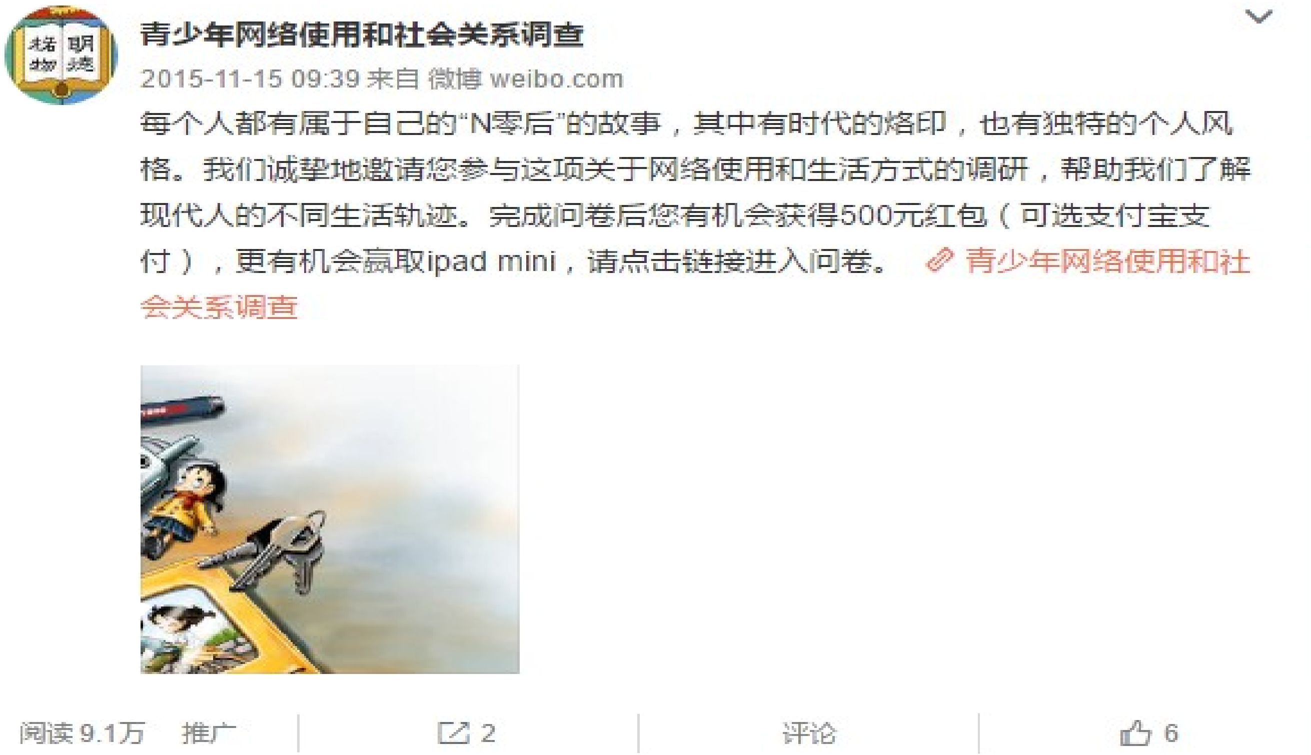

青少年网络使用和社会关系调查

2015-11-15 09:39 来自 微博 weibo.com

每个人都有属于自己的“N零后”的故事，其中有时代的烙印，也有独特的个人风格。我们诚挚地邀请您参与这项关于网络使用和生活方式的调研，帮助我们了解现代人的不同生活轨迹。完成问卷后您有机会获得500元红包（可选支付宝支付），更有机会赢取ipad mini，请点击链接进入问卷。 青少年网络使用和社会关系调查

阅读 9.1万 推广 | 2 | 评论 | 6
